# Multiparametric Ultrasound Examination in Tumor-Like Formations of the Ovaries

**DOI:** 10.25122/jml-2020-0090

**Published:** 2020

**Authors:** Iryna Dmytrivna Stasiv, Valeryan Mykolayovych Ryzhyk, Vasyl Hryhorovych Mishchuk, Petro Fedorovych Dudiy, Tetyana Ivanivna Salyzhyn

**Affiliations:** 1. Department of Radiology and Radiation Medicine, Ivano-Frankivsk National Medical University, Ivano-Frankivsk, Ukraine; 2. Department of General Practice (Family Medicine), Physical Rehabilitation and Sports Medicine, Ivano-Frankivsk National Medical University, Ivano-Frankivsk, Ukraine; 3. Department of Internal Medicine No. 1, Clinical Immunology and Allergology, E.M. Neyka, Ivano-Frankivsk National Medical University, Ivano-Frankivsk, Ukraine

**Keywords:** Ovarian tumor-like formations, ultrasound examination, compression elastography

## Abstract

Properly diagnosed tumor-like formations of the ovaries facilitate the correct selection of patients who may not require surgery, or choose surgery with minimal access if such intervention is required. Subjective assessment of the features of tumor-like formations with the help of ultrasound diagnostics, including compression elastography, proved to be highly effective in the differential diagnosis of bulky ovarian formations. All tumor-like formations have their sonographic features that allow making a reliable diagnosis of a particular formation. The article reveals data on the diagnostic significance of multiparametric ultrasound imaging in the detection of ovarian tumor-like formations. A detailed sonographic picture of tumor-like formations in B-mode, color, and pulse Doppler mode and compression sonoelastography mode was analyzed. This examination was especially relevant for women of reproductive age, as it depended on the further tactics of treatment of each patient. For all types of tumor-like formations ovaries, a qualitative feature was determined - elastotype on the Ueno scale and the index of stiffness (Strain Ratio) - a quantitative indicator. Follicular cysts, endometrioid and periovarian cysts were found to belong to the 0 elastotype. Cysts of the corpus luteum belonged to the II elastotype on the Ueno scale. The lowest values of the stiffness index were seen in follicular and periovarian cysts, and the highest value was observed in endometrioid cysts. Our results have shown that ultrasound examination of ovarian tumors is an accurate and highly informative method.

## Introduction

Space-occupying tumor-like masses in the ovaries are one of the most frequently identified formations in gynecological practice. Early differential diagnostics is a key factor in the medical management of every woman [1]. This pathology is especially relevant for women of reproductive age. In most cases, the presence of cysts is associated with infertility problems. In many women, there is a pain syndrome and ovarian-menstrual cycle irregularities, leading to a necessity of consultation with obstetrician-gynecologists or reproductive specialists [2-4].

The peculiarities of ovarian tumor-like formations and differential diagnosis between benign, tumor-like and malignant formations are important both to reduce excessive anxiety in patients and to decide on the choice of treatment tactics options, which, in turn, increases and improves patient survival and reproductive function in women [5, 6].

Each menstrual cycle ends with the development of one Graafian follicle. It protrudes above the ovary’s surface, there is a rupture of its wall, and the egg cell enters the abdominal cavity. When the development of the follicle, and function of ovulation and corpus luteum is disturbed, pathological formations in the form of cysts are possible [7].

Tumor-like formations of the ovaries have different ultrasound characteristics [8]. Some cystic formations may be complex cysts with exfoliation, hemorrhage, or thick walls. Differential diagnosis between tumor-like, benign, and malignant ovarian lesions is quite difficult and transvaginal, and transabdominal ultrasonography becomes a tool of primary imaging [8, 9]. According to the classification of the International Ovarian Tumor Analysis group, multilocular cysts with a solid component have the highest risk of malignancy [8]. Multiparametric ultrasound examinations allow making a more accurate differential diagnosis between tumor-like, benign, and malignant masses.

The grayscale ultrasonography in B-mode of ovarian neoplasms is known to have similar characteristics often, so this modality of ultrasound diagnosis is quite subjective. Differential diagnosis of ovarian formations is facilitated by Doppler regimens. It has been proven that benign and malignant ovarian masses have intranodular blood flow, and tumor-like ones are characterized by extranodular blood flow [10-12]. However, there are cases when Doppler regimens do not register low-velocity blood flow in ovarian masses. In this case, it is advised to move on to the next ultrasound modality – sonoelastography, which serves as a new method of assessing tissue stiffness and is widely used to distinguish between benign and malignant pathology of the mammary and thyroid glands. It is becoming an increasingly popular component of gynecological ultrasonography. To conduct this study, it was necessary to switch the scanner to real-time sonoelastography [13-18]. The advantage of using this method in the diagnostic algorithm of patients with ovarian tumor-like formations is its lower cost compared to magnetic resonance imaging (MRI) [19].

Our study aims to improve the diagnosis of tumor-like formations of the ovaries using multiparametric ultrasonography with particular attention to real-time compression sonoelastography, which gives additional characteristics of the elasticity of these pathological conditions.

Despite the frequent use of magnetic resonance imaging and computed tomography to characterize ovarian pathology, qualitative images of ovarian masses can be obtained using multiparametric ultrasonography, which is based on the use of standard B-mode, color Doppler mapping, energy and spectral Doppler mode and thus simplifying the process of diagnosing this pathology [8, 9].

## Material and Methods

The mean age of patients was (34.77 ± 2.07) years. The most common complaint of the examined patients was pain syndrome – 42 (84%) cases and menstrual irregularities – 34 (68%) cases. Bloody discharge and infertility were diagnosed in 21 (42%) and 10 (20%) cases, respectively. According to the medical history of the patient, nonsteroidal anti-inflammatory drugs and antibiotics were taken by patients before the diagnostic search. This group of patients showed an elevated mean body mass index, which was (24.81 ± 1.23).

A dynamic ultrasound examination of 50 women with suspected ovarian tumor-like formations was performed. The examination was performed on an ALOCA ARIETTA 60 system (Hitachi), using a sector sensor, with a 3.5 MHz frequency for transabdominal examination and an endocavity sensor, 8 MHz – for transvaginal examination.

Firstly, all women underwent a standard transvaginal ultrasound examination in B-mode using color, energy, and pulsed Doppler techniques. After that, to determine the density of the detected mass, compression elastography was performed. The study was conducted in real-time. The area of interest was marked as ROI (region of interest), and the parameters of sonoelastography were optimized: intensity, mechanical index, and the optimal compression parameters for the study area were controlled using a scale or graph on the monitor screen. For proper elastography, the examination area also involved at least 3/4 of the unaltered reference tissue. The determination of the stiffness index is based on a comparative analysis of the density of normal and pathologically altered tissue, and the elastographic image of the affected ovary was compared with the elastographic image of the unaltered part of the ovary. All the obtained results in the form of static images were stored in the memory of the device, which enabled their digital interpretation.

Qualitative assessment of tumor-like formations density was performed using the classification of elastotypes on the UENO scale. All images that can be attributed to 0, 1 and 2 elastotypes correspond to benign formations and those belonging to the 3 elastotype correspond to conditionally benign. The images obtained in 4 and 5 elastotypes were characteristic of malignant neoplasms.

At the same time, not only the qualitative characteristics of the lesion foci were evaluated, but also the quantitative characteristics, i.e., the coefficient of tissue deformation - strain ratio values - were determined. This ratio allows estimating the ratio of the ovarian formation density to the density of the surrounding unaltered tissues.

Criteria for inclusion into the study were the presence of informed consent for ultrasound examination, suspicion of ovarian tumor-like formations, the reproductive age of patients (18-50 years).

The exclusion criteria were patients under 18 years of age and women over 50 years (during menopause), pregnant women, and patients with ovarian apoplexy.

The obtained data were processed using the STATISTICA (Stat Soft Statistic v.6.0) software package. The reliability of the obtained results was evaluated by the bidirectional non-parametric Student’s t-test. The threshold level of statistical significance was taken as p<0.05.

## Results

According to the research results, in 39 (78%) examined patients, tumor-like formations were detected for the first time, and in 11 (12%) – repeatedly. At the same time, the reproductive function was maintained in 22 (44%) women, and 28 (56%) have not given birth to children. Surgery due to ovarian tumor-like formations was performed in 13 (26%) women, including 8 (61.5%) patients with endometriomas and 5 (38.5%) with paraovarian cysts. Bimanual gynecological examination revealed a painful three-dimensional ovarian formation in 33 (66%) women and an increase in uterine size in 34 (68%) cases. Signs of inflammation in the cytological examination of a smear from the cervix were seen in 24 (48%) women.

**Figure 1:**
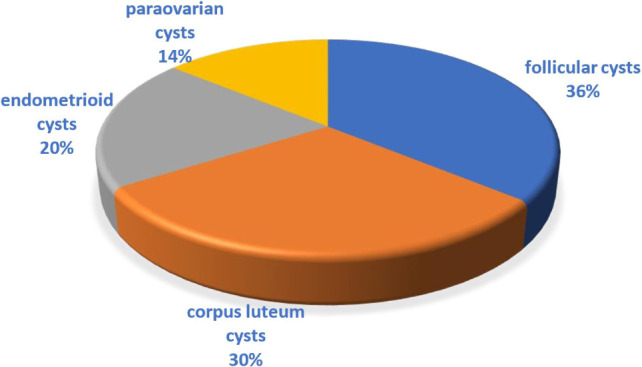
The structure of the distribution of tumor-like formations of the ovaries.

As a result of dynamic observation of patients, the following types of tumor-like formations of ovaries were revealed (Figure 1).

**Figure 2:**
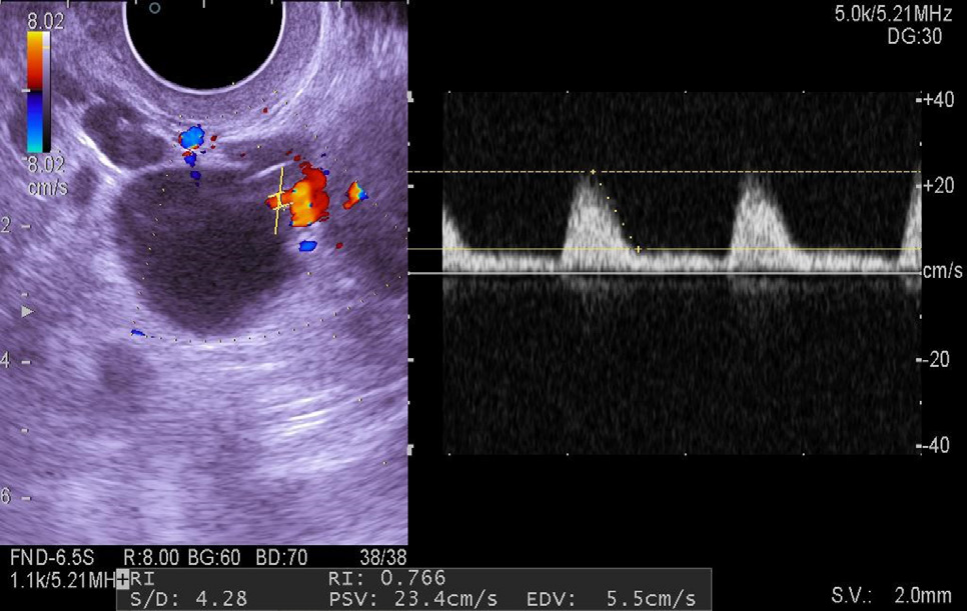
Sonogram of a follicular cyst with Doppler ultrasonography.

As can be seen from the data shown in the diagram, follicular and corpus luteum cysts were most often diagnosed, and endometrioid and paraovarian cysts were somewhat less frequently diagnosed. Peculiarities of follicular and paraovarian cysts are as follows: anechogenic formation, with a clear, smooth contour, regular inner surface, homogeneous content, unaltered ovarian tissue was detected on the periphery of the cyst, peripheral blood supply (Figure 2).

**Figure 3:**
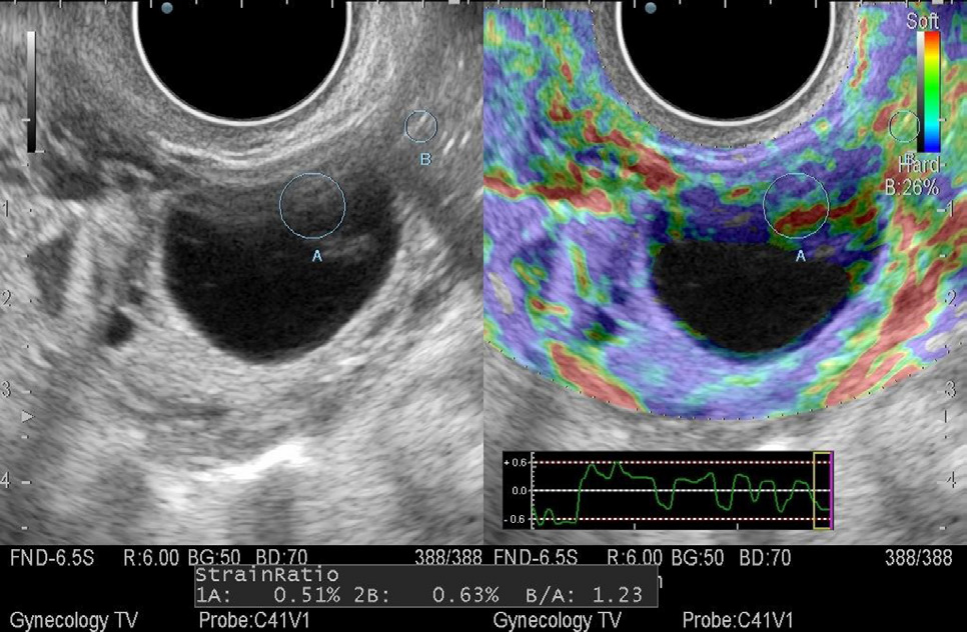
Sonoelastogram of a follicular cyst.

Mode of sonoelastography: a limitation for this type of study were large anechogenic formations, the size of which exceeded 40 mm, because such formations are not mapped at all, or is determined only in the upper third of the formation. With a smaller size, cysts were mapped in blue-green-red colors, which corresponds to the 0 elastotype, and their stiffness index ranged from 0.219 to 1.23 units (Figure 3).

**Figure 4:**
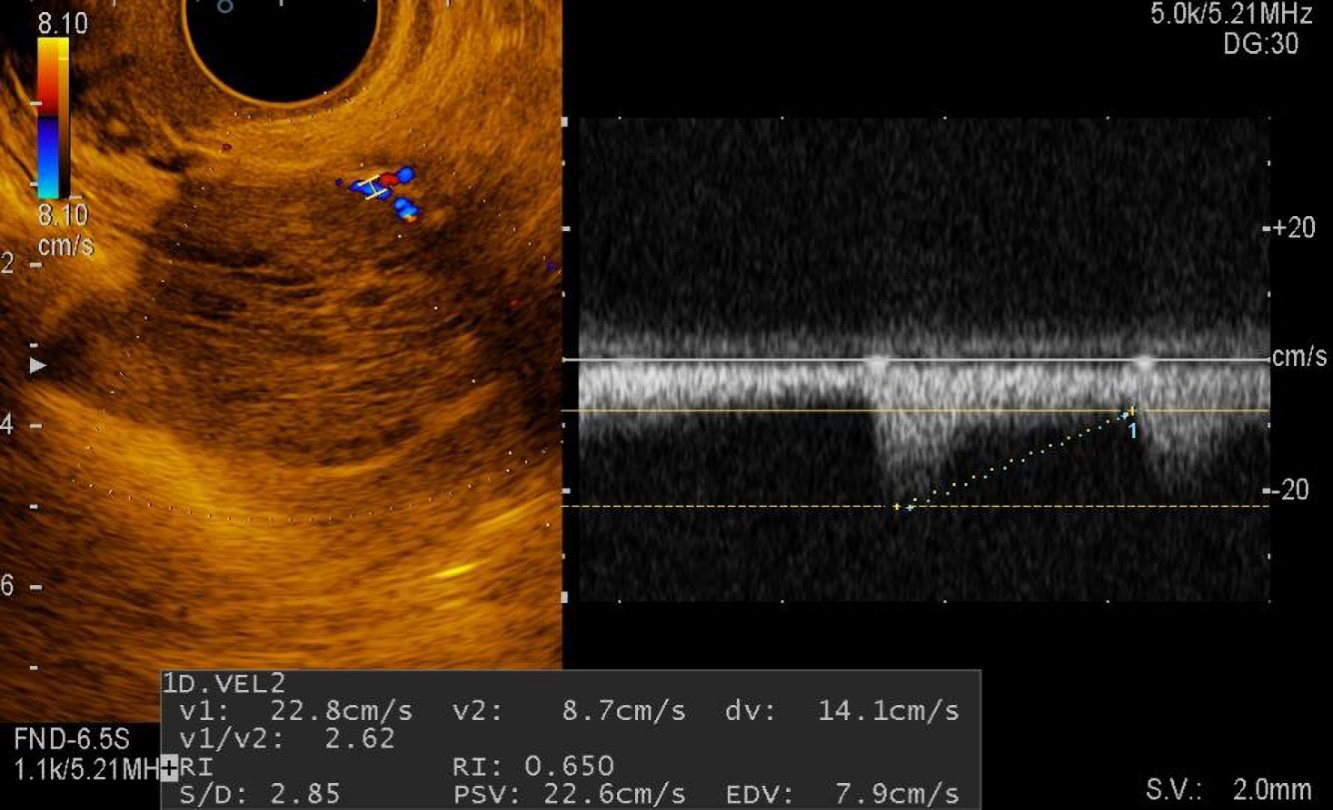
Sonogram of a corpus luteum cyst with Doppler ultrasonography.

**Figure 5:**
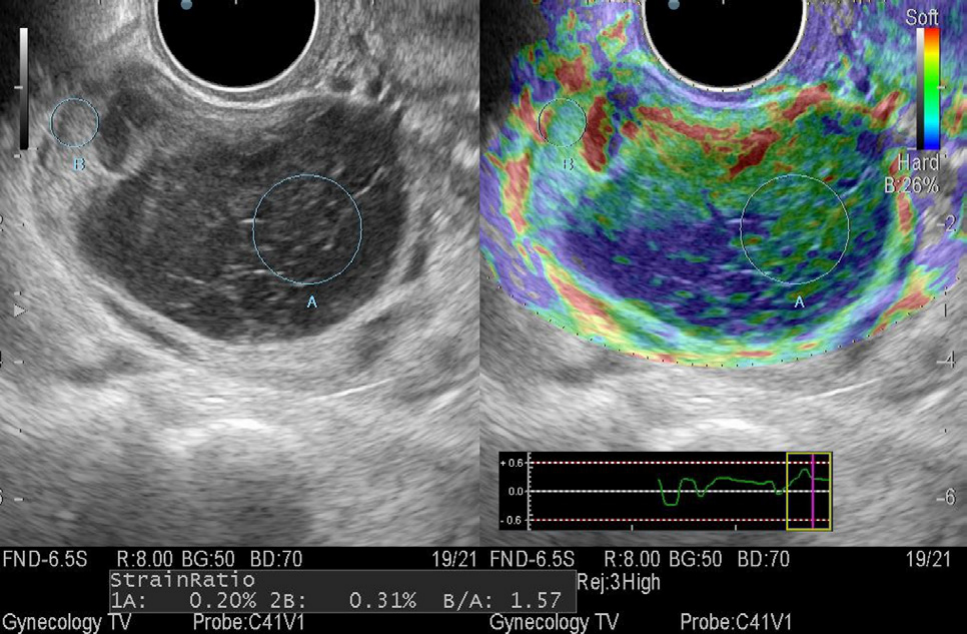
Sonoelastogram of a corpus luteum cyst.

In 15 women (30%), heterogeneous formations, with smooth, clear contours, inhomogeneous due to arachnoid septa, and unaltered ovarian tissue on the periphery were detected. In these formations, the blood supply was determined at the periphery, which, in turn, allows differentiation of hemorrhage into the cyst from a solid component (Figure 4). These formations in the mode of sonoelastography are mapped in green with admixtures of blue, which is characteristic of the 2 elastotype (Figure 5). Their index of stiffness (Strain Ratio) ranges from 0.98 to 3.08 relative units.

**Figure 6:**
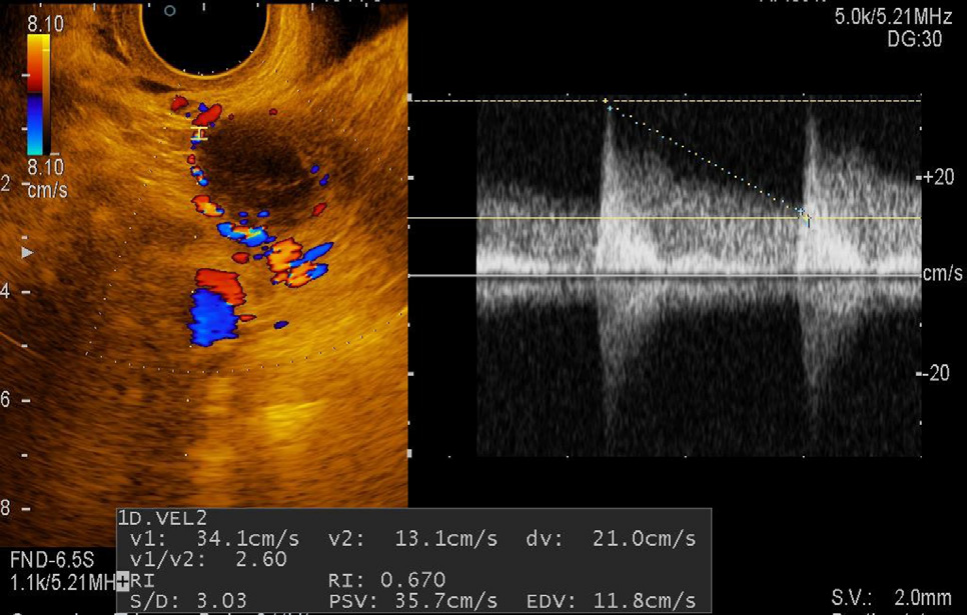
Sonogram of an endometrioid cyst with Doppler ultrasonography.

**Figure 7:**
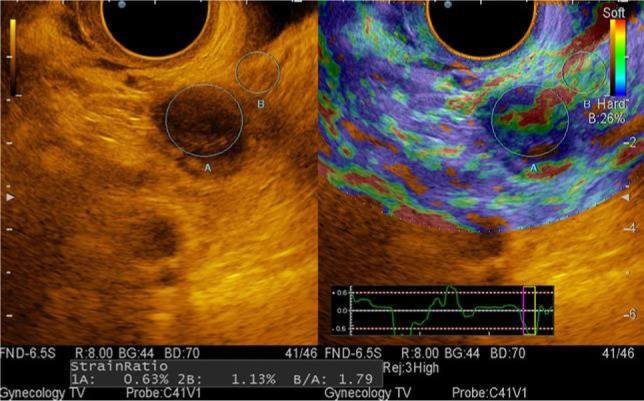
Sonoelastogram of an endometrioid cyst.

In 10 women, there were formations with fine-grained content, with clear, smooth contours. However, in 5 cases, the inner wall was irregular, papillary growths were not detected, and extranodular blood flow was seen in all cases (Figure 6). When choosing the mode of sonoelastography, all formations were mapped in blue-green-red colors, which corresponds to the 0 elastotype. The stiffness index strain ratio ranged from 0.819 to 3.23 relative units (Figure 7).

Quantitative indices of Doppler ultrasonography in women with tumor-like formations of the ovaries are characterized by an average value of Vmax (39.08 ± 2.51) cm/sec, 2.16 ± 0.061 Standard Deviation (SD) relative units, 0.59 ± 0.01 Resistive Index (RI) relative units.

## Discussion

This study was performed for a detailed sonographic description of the ovarian formations, taking into consideration the frequency of tumor-like diseases, the relevance of their rapid and cheap diagnosis, the lack of data in the available sources on multiparametric ultrasound examination with special attention to sonoelastography.

A detailed ultrasound picture of benign and tumor-like formations of the ovaries in the B-mode was described by Ionescu et al. in 2018. However, they did not use compression elastography in their studies. Characteristics of sonoelastographic imaging of ovarian tumor-like formations were also described by Gazhonova et al. [20]. The common feature of our studies is that uncomplicated follicular and endometrioid cysts corresponded to the 1 elastotype on the UENO scale and there was no significant difference in the indices of strain ratio. However, in the suggested research, there is no sonoelastographic description of complicated and corpus luteum cysts, and there is no description of Doppler imaging of ovarian tumor-like conditions. Doppler analysis of these formations is described by Ashrafyan et al. [21]. They revealed that average Vmax indices were slightly lower than our data and equaled to 27.5 ± 4.8 relative units, and the strain ratio indices had no significant differences.

Compression elastography has become a helpful tool for ultrasound diagnosis of three-dimensional formations. A potential application of this technique is its association with grayscale and Doppler modes, i.e., multiparametric ultrasound examination, which is helpful in complicated diagnostic cases.

## Conclusions

In the structure of tumor-like pathology of the ovaries, the frequency of follicular cysts is 36%, corpus luteum cysts – 30%, endometrioid cysts – 20% and paraovarian cysts – 14%.

Ultrasound examination, as the least invasive method of diagnosis, allows diagnosing follicular and endometrioid cysts of the ovary with accuracy up to 100%.

Since ovarian tumor-like formations in most cases occur in women of fertile age, the maximum efforts of gynecologists and reproductive specialists should be aimed at timely diagnosis and treatment of this pathology, which will preserve the reproductive capacity and reduce the percentage of unnecessary surgical invasions. The innovative technology of sonoelastography provides qualitatively new information about the elasticity of tissues and allows assessing the stiffness of ovarian tumor-like formations.

Compression sonoelastography can be recommended in the algorithm of a comprehensive ultrasound examination of the ovaries.

## Conflict of Interest

The authors declare that there is no conflict of interest.

## References

[1] Muto M. Patient education: Ovarian cysts (Beyond the Basics). Section Editors: Barbara Goff, William J Mann.

[2] Zvarych LI., Lutsenko NS, Shapoval OS (2015). Frequency of functional ovarian cysts in women of reproductive age in the structure of gynecological pathology. Modern medical technologies.

[3] Shapoval OS. (2016). Clinical and sonological features in tumor-like formations of the ovaries in women of reproductive age. Women’s health.

[4] Shapoval OS. (2016). Clinical and sonological features in tumor-like formations of the ovaries in women of reproductive age. Women’s health.

[5] Salihoglu KN., Dilbaz B, Cırık DA (2016). Short-term impact of laparoscopic cystectomy on ovarian reserve tests in bilateral and unilateral endometriotic and nonendometriotic cysts. J Minim Invasive Gynecol.

[6] Ionescu CA., Matei A, Navolan D, Dimitriu M, Bohâltea R, Neacsu A, Ilinca C, Ples L (2018). Correlation of ultrasound features and the Risk of Ovarian Malignancy Algorithm score for different histopathological subtypes of benign adnexal masses. Medicine (Baltimore).

[7] Gerasimova TV. (2015). Optimization of diagnosis and treatment of functional ovarian cysts. Reproductive endocrinology.

[8] Choi JI., Park SB, Han BH, Kim YH, Lee YH, Park HJ (2016). Imaging features of complex solid and multicystic ovarian lesions: proposed algorithm for differential diagnosis. Clin Imaging.

[9] Dias DS., Bueloni-Dias FN, Delmanto A, Tonon AF, Tayfour NM, Traiman P (2015). Clinical management of incidental findings on pelvic adnexal masses. Rev Assoc Med Bras.

[10] Jokubkiene L., Sladkevicius P, Valentin L (2007). Does three-dimensional power Doppler ultrasound help in discrimination between benign and malignant ovarian masses?. Ultrasound Obstet Gynecol.

[11] Guerriero S., Ajossa S, Risalvato A, Lai MP, Mais V, Angiolucci M (1998). Diagnosis of adnexal malignancies by using color Doppler energy imaging as a secondary test in persistent masses. Ultrasound Obstet Gynecol.

[12] Tailor A., Jurkovic D, Bourne TH, Collins WP, Campbell S (1997). Sonographic prediction of malignancy in adnexal masses using multivariate logistic regression analysis. Ultrasound Obstet Gynecol.

[13] Gweon HM., Youk JH, Son EJ, Kim JA (2013). Clinical application of qualitative assessment for breast masses in shear-wave elastography. Eur J Radiol.

[14] Yoon JH., Ko KH, Jung HK, Lee JT (2013). Qualitative pattern classification of shear wave elastography for breast masses: how it correlates to quantitative measurements. Eur J Radiol.

[15] Lacout A., Chevenet C, Thariat J, Figl A, Marcy PY (2013). Qualitative ultrasound elastography assessment of benign thyroid nodules: patterns and intraobserveracquisition variability. Indian J Radiol Imaging.

[16] Itoh A., Ueno E, Tohno E, Kamma H, Takahashi H, Shiina T (2006). Breast disease: clinical application of US elastography for diagnosis. Radiology.

[17] Garg S., Kaur A, Mohi J, Kanwal Sibia P. (2017). Evaluation of IOTA simple ultrasound rules to distinguish benign and malignant ovarian tumours. J Clin Diagn Res.

[18] Borsukov AV., Morozova TG, Kovalev AV, Kazakova OP, Mamoshin AV, Smyslenova MV, Vasilieva Yu.N., Sinyukova GT, Danzanova T.Yu., Busko EA, Rakhimzhanova RI, Fazylova SA (2015). Trends in the development of compression sonoelastography of superficial organs and endosonography in the framework of standardization of the technique. Bulletin of New Medical Technologies.

[19] Onur MR., Simsek BC, Kazez A (2011). Sclerosing stromal tumor ovary ultrasound elastography MRI findings preoperative diagnosis. J Med Ultrason.

[20] Gajonova VE., Churkina SO (2008). Clinical application of a new method of sonoelastography in gynecology. Kremlin Medicine.

[21] Ashrafyan LA., Babaeva NA, Antonova IB, Ivashina SV, Lustik AV, Aleshikova OI, Gerfanova EV, Dobrenko AA (2015). Ultrasonic criteria for early diagnosis of ovarian cancer of the female reproductive system.

